# Prediction of Microvascular Invasion of Hepatocellular Carcinoma Based on Contrast-Enhanced MR and 3D Convolutional Neural Networks

**DOI:** 10.3389/fonc.2021.588010

**Published:** 2021-03-04

**Authors:** Wu Zhou, Wanwei Jian, Xiaoping Cen, Lijuan Zhang, Hui Guo, Zaiyi Liu, Changhong Liang, Guangyi Wang

**Affiliations:** ^1^ School of Medical Information Engineering, Guangzhou University of Chinese Medicine, Guangzhou, China; ^2^ Shenzhen Institutes of Advanced Technology, Chinese Academy of Sciences, Shenzhen, China; ^3^ Department of Optometry, Guangzhou Aier Eye Hospital, Jinan University, Guangzhou, China; ^4^ Department of Radiology, Guangdong Provincial People’s Hospital, Guangdong Academy of Medical Sciences, Guangzhou, China

**Keywords:** hepatocellular carcinoma, microvascular invasion, convolutional neural network, deeply supervised network, contrast-enhanced MR

## Abstract

**Background and Purpose:**

It is extremely important to predict the microvascular invasion (MVI) of hepatocellular carcinoma (HCC) before surgery, which is a key predictor of recurrence and helps determine the treatment strategy before liver resection or liver transplantation. In this study, we demonstrate that a deep learning approach based on contrast-enhanced MR and 3D convolutional neural networks (CNN) can be applied to better predict MVI in HCC patients.

**Materials and Methods:**

This retrospective study included 114 consecutive patients who were surgically resected from October 2012 to October 2018 with 117 histologically confirmed HCC. MR sequences including 3.0T/LAVA (liver acquisition with volume acceleration) and 3.0T/e-THRIVE (enhanced T1 high resolution isotropic volume excitation) were used in image acquisition of each patient. First, numerous 3D patches were separately extracted from the region of each lesion for data augmentation. Then, 3D CNN was utilized to extract the discriminant deep features of HCC from contrast-enhanced MR separately. Furthermore, loss function for deep supervision was designed to integrate deep features from multiple phases of contrast-enhanced MR. The dataset was divided into two parts, in which 77 HCCs were used as the training set, while the remaining 40 HCCs were used for independent testing. Receiver operating characteristic curve (ROC) analysis was adopted to assess the performance of MVI prediction. The output probability of the model was assessed by the independent student’s t-test or Mann-Whitney U test.

**Results:**

The mean AUC values of MVI prediction of HCC were 0.793 (p=0.001) in the pre-contrast phase, 0.855 (p=0.000) in arterial phase, and 0.817 (p=0.000) in the portal vein phase. Simple concatenation of deep features using 3D CNN derived from all the three phases improved the performance with the AUC value of 0.906 (p=0.000). By comparison, the proposed deep learning model with deep supervision loss function produced the best results with the AUC value of 0.926 (p=0.000).

**Conclusion:**

A deep learning framework based on 3D CNN and deeply supervised net with contrast-enhanced MR could be effective for MVI prediction.

## Introduction

Hepatocellular carcinoma (HCC) has become the fourth most common cause of cancerous death in the world (1). Microvascular invasion (MVI) of HCC has been shown to be a key predictor of recurrence and poor prognosis. Furthermore, preoperative knowledgement of MVI of HCC can be helpful in deciding treatment strategy and patient management (2). However, MVI is not similar to the macrovascular invasion, which can be evaluated by radiologic images. The gold standard of MVI information is generally determined by the histopathological characteristics of HCC lesions (3, 4). Therefore, it is desirable to develop a preoperative method for MVI prediction with non-invasive assessments.

Many studies focus on imaging findings of preoperative imaging to predict MVI of HCC. Numerous clinical features, including tumor size, edge smoothness, capsule, tumor peripheral enhancement, multifocality, apparent diffusion coefficient (ADC), alpha-fetoprotein (AFP), 18F-deoxyglucose (18F-FDG), have been shown to be helpful for the preoperative prediction of MVI (5–7). However, these radiologic features for MVI prediction were shown in inconsistent conclusions in different studies (8). Recently, Wei et al. (9) prospectively evaluated the potential role of intra voxel incoherent motion (IVIM), and demonstrated that the D value obtained by the IVIM model was better than the ADC used to evaluate the MVI of HCC, while it is reported that the tumor edge, enhancement mode, tumor capsule, and enhancement around the tumor have no predictive effect on the MVI in MR imaging characteristics. In addition, those radiologic findings are generally limited by individual experience, which may have inter-observer errors and be insufficient for MVI prediction.

Recently, radiomics has been widely utilized as non-invasive predictive biomarker in clinical practice (10), which has also been successfully applied in MVI prediction (10). Peng et al. (11) proposed an imaging radiomics model to predict the MVI risk of HCC before surgery, in which 980 candidate imaging radiomics features were obtained from the arterial and portal vein phases and significantly correlated with the MVI status. Bakr et al. (12) also proposed a non-invasive imaging radiomics model, including 717 quantitative features from arteries, portal veins, and delayed phases of contrast-enhanced CT images for MVI prediction of HCC. Feng et al. (13) developed a radiomics model for preoperative MVI prediction. Ma et al. (14) also proposed and validated an imaging radimoics model to use contrast-enhanced CT images to predict preoperative MVI in HCC patients. Xu et al. (15) recently analyzed the contrast-enhanced CT based on radiomics analysis to predict MVI and outcome in HCC, and demonstrated that the combination of radiological and imaging radiomics features could produce better performance in predicting MVI. In addition, there also have been some reports about radiomics predicting MVI based on ultrasound images (16, 17). As pointed out by the recent study (8), researchers currently construct radiomics models based on single modality image data, and the use of multimodality for MVI prediction has not to be investigated. In addition, due to the sensitivity of imaging radiomics features to acquisition methods and reconstruction parameters, the imaging radiomics features are very unreliable to be widely used in clinical practice (18).

The deep features obtained by direct learning from medical imaging data have been shown to be superior to traditional imaging radiomics features, which have been widely used in medical imaging analysis and clinical lesion characterization (19). As a new feature descriptor, deep feature is a result of autonomous learning compared with the traditional morphological texture feature, which avoids the typical drawback in the design of manual features (20, 21). Convolutional neural network (CNN) is currently the most successful type of deep learning model in image analysis (22). It has exhibited remarkably high performance in the diagnosis of liver fibrosis and liver masses (23, 24). Therefore, deep feature derived from CNN may be advantageous for tumor characterization. Inspired by the work of deep learning with multiple modalities combined to generate complementary improvements than single modality (25), it can be anticipated that the relationship between multiple phases of contrast-enhanced images can be learned by deep convolutional networks with multiple modalities and the learned deep features presentation from multiple phases of contrast-enhanced images may be useful for MVI prediction.

To this end, we preliminarily propose a deep learning network structure based on 3D CNN, which extracts HCC discriminant image features from multiphase images of contrast-enhanced MR for MVI prediction. Specifically, we first carefully evaluate the representation performance of 3D deep features in each phase, and then use feature concatenation to take advantage of the discriminative features from contrast-enhanced MR. Finally, we design a loss function for deep supervision of features from different phases of contrast-enhanced MR to achieve the best MVI prediction.

## Material and Methods

### Dataset and Preprocessing

#### Study Population

**Figure 1:**
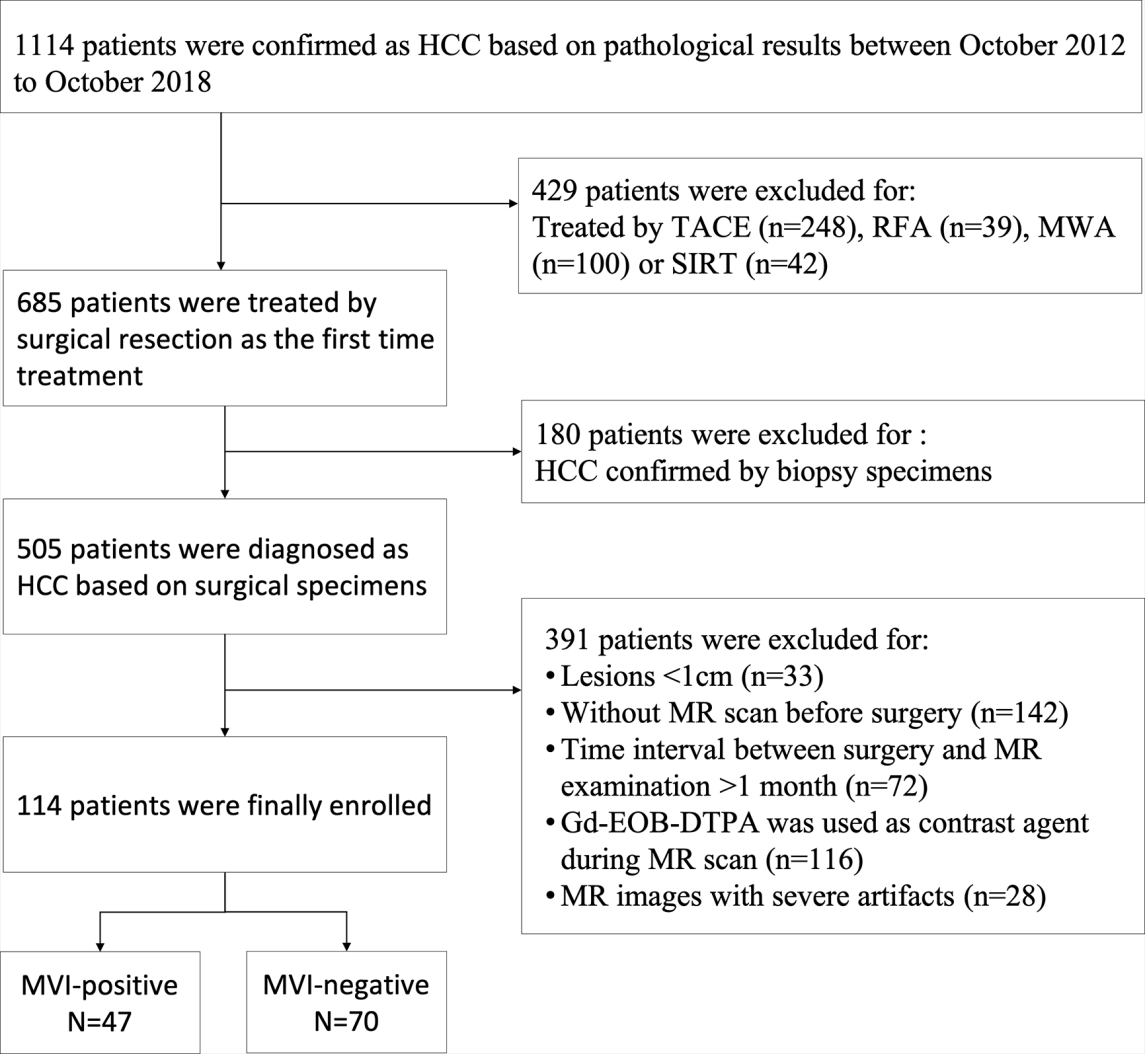
Flow chart of patients’ recruitment for the study. TACE, transcatheter arterial chemoembolization; RFA, radiofrequency ablation; MWA, microwave ablation; SIRT, selective internal radiation therapy.

The present study has been approved by the local institutional review board, and the patient’s informed consent has also been obtained. In the time from October 2012 to October 2018, a total of 1114 consecutive patients were diagnosed as HCCs based on pathological results at our hospital. The inclusion criteria for this study were as follows (**Figure 1**): a) without prior treatment including microwave ablation (MWA), selective internal radiation therapy (SIRT), radiofrequency ablation (RFA), or transcatheter arterial chemoembolization (TACE); b) HCC was confirmed by evaluating surgical specimens; c) MR imaging examination should be performed no earlier than 1 month before the surgery. In addition, the exclusion criteria of cases are as follows: a) without MR imaging examination before hepatectomy; b) without contrast-enhanced MR imaging examination before hepatectomy; c) small HCCs lesions less than 10mm in diameter; d) MR images with severe artifacts. Specifically, we excluded small HCC with tumor diameter smaller than 10mm because of the difficulty in determining its 3D region on MR. Furthermore, surgical resection is rarely used for such small lesions and the pathological information is often unavailable.

#### MRI Protocol

Gd-DTPA-enhanced MR imaging of patients were conducted by two kinds of 3.0T MR scanners, including Signa Excite HD 3.0T (GE Healthcare, Milwaukee, WI, USA) with breath-hold axial LAVA (liver acquisition with volume acceleration, LAVA) protocol and Achieva 3.0T (Philips Medical Systems, Netherlands) with axial e-THRIVE (enhanced T1 high-resolution isotropic volume excitation sequence, e-THRIVE) protocol. The bolus injection rate of the contrast agent Gd-DTPA (Magnevist, Bayer-Schering Pharma AG) was set to 2.5ml/s, and the contrast dose of each patient was 0.025mmol/kg body weight (0.1 ml/kg), and 15 ml of saline were subsequently flushed through the power injector at a rate of 2 ml/s for each patient. The contrast-enhanced MR image consisted of images of arteries, portal veins, and delayed phases. Ideally, contrast-enhanced MR images could be obtained at 25–30, 45, and 70 s after Gd-DTPA injection during breath-holding. The parameters of the two different scanners were shown in **Table 1**.

**Table 1:** Parameters of two MRI scanners.

Parameters	GE Signa Excite	Philips Achieva
Echo time (TE) (ms)	1.95	1.944
Repetition time (TR) (ms)	4.25	4.02
Field of view (FOV) (mm)	741´380	649´330
Slice thickness (mm)	2.2	6
Slice gap (mm)	1	3
Flip angle (degrees)	90	10

#### Clinical and Pathological Characteristics

**Table 2** summarizes the baseline clinical and pathological characteristics of all patients. The pathological diagnosis of HCC was based on surgically resected specimens, and the histological information of HCC was retrieved from archived clinical histology reports. The gold standard for diagnosis of MVI was pathological diagnosis which was based on surgically resected specimens. MVI positive was defined as tumor cells in blood vessels lined by endothelium that was visible only under the microscope. In addition, other pathological features were also evaluated, such as Edmondson-Steiner tumor grade.

**Table 2:** Clinical characteristics of patients in training and validation cohorts.

Characteristics	Training		*P*_intra_	Validation		*P*_intra_	*P*_inter_
	MVI-negative (n=47)	MVI-positive (n=30)		MVI-negative (n=23)	MVI-positive (n=17)		
**Patient characteristics**
Age (y)*	56.94 ± 13.37	51.57 ± 11.48	0.046	52.61 ± 13.94	50.12 ± 10.21	0.518	0.141
**No. of male patients**	42 (89.4)	25 (83.3)	0.675	22 (95.7)	17 (100)	1.000	0.131
**No. of patients with hepatitis B virus infection**	41 (87.2)	30 (100.0)	0.109	22 (95.7)	14 (82.4)	0.394	0.955
Total bilirubin (µmol/L)*	16.96 ± 9.64	17.37 ± 6.60	0.590	20.57 ± 7.82	17.18 ± 8.35	0.197	0.059
Albumin (g/L)*	38.53 ± 7.00	37.97 ± 8.51	0.777	38.13 ± 8.31	37.29 ± 5.68	0.967	0.691
Alanine aminotransaminase level (U/L)*	50.87 ± 82.23	58.93 ± 51.09	0.090	41.04 ± 19.59	52.29 ± 46.51	0.805	0.767
Aspartate aminotransaminase level (U/L)*	50.02 ± 46.26	57.37 ± 50.52	0.242	45.17 ± 25.21	63.88 ± 45.41	0.184	0.562
*γ*-Glutamyltransferase (U/L)*	77.17 ± 77.16	96.03 ± 81.09	0.088	59.09 ± 47.99	75.94 ± 37.18	0.090	0.696
Prothrombintime (s)*	15.87 ± 2.36	14.83 ± 1.93	0.055	14.83 ± 2.29	15.35 ± 2.03	0.627	0.576
**Tumor-related factors**							
α-Fetoprotein level (ng/ml)*	2,375.94 ± 9,035.77	6,093.30 ± 16,365.98	0.045	165.30 ± 336.91	11,308.65 ± 20,057.58	0.030	0.179
Carbohydrate antigen 19-9 (U/ml)*	37.79 ± 83.12	26.90 ± 29.34	0.942	23.61 ± 20.44	22.41 ± 21.65	0.945	0.739
**Nodules characteristics**						
Nodules long diameter (mm)*	46.44 ± 31.99	60.20 ± 33.54	0.039	41.61 ± 31.24	73.18 ± 40.18	0.022	0.984
**No. of patients with portal-vein invasion**	1 (2.1)	1 (3.3)	1.000	0 (0)	3 (17.6)	0.137	0.446
**No. of patients with multiple nodules**	10 (21.3)	11 (36.7)	0.139	5 (21.7)	6 (35.3)	0.555	0.979
**Presence of hemorrhage**	28 (59.6)	11 (36.7)	0.050	17 (73.9)	7 (41.2)	0.037	0.336
**Pathologic findings**							
**Edmondson-Steiner grade**
**G1–G2**	24 (51.1)	10 (33.3)	0.193	13 (56.5)	5 (29.4)	0.046	0.905
**G3–G4**	20 (42.6)	16 (53.3)		8 (34.8)	12 (70.6)		

^*^Data are means ± standard deviation.

#### Volumetric Region Extraction and Data Augmentation

All MR images were transferred to a workstation (Precision T7610; Dell Inc, Austin, USA) for postprocessing. A radiologist with 15 years of experience and another with 5 years of experience separately analyzed the tumor regions of interest (ROI) in medical images. The volumetric tumor regions were manually extracted by the in-house software implemented by Matlab (26). The training process of deep learning networks usually requires thousands of samples to complete. Data augmentation used the image resampling method (27) to extract more 3D samples from the current limited tumor sample area. First, the three-dimensional cube area of the tumor was manually extracted, which was placed at the center of mass of the 3D HCC. Note that the volumetric region contains the whole tumor and it also includes the area of non-tumor liver that outsides the tumor boundary to inside the volumetric cube. Due to the different sizes of tumors, all extracted cube tumor regions were normalized to a preset size (for example, 32×32×32), and many relatively small cubes (for example, 16×16×16) overlapping blocks can be extracted as the sample to train the network. Specifically, a 3D block with a size of 16×16×16 was centered on the centroid of the tumor, and then a translation (2 pixels) was performed along the axial, coronal, and sagittal directions within the normalized cube. There were a total of Na * Nc * Ns translations, where Na, Nc, and Ns were the times of translations along the axial, coronal, and sagittal directions, respectively. Na, Nc, and Ns were set to 7 in this work, and finally 343 block samples were obtained from each 3D tumor. Note that data augmentation was only performed on the training tumor data, not on the test tumor data.

### The Proposed Method

#### The Overview of the Proposed Framework

**Figure 2:**
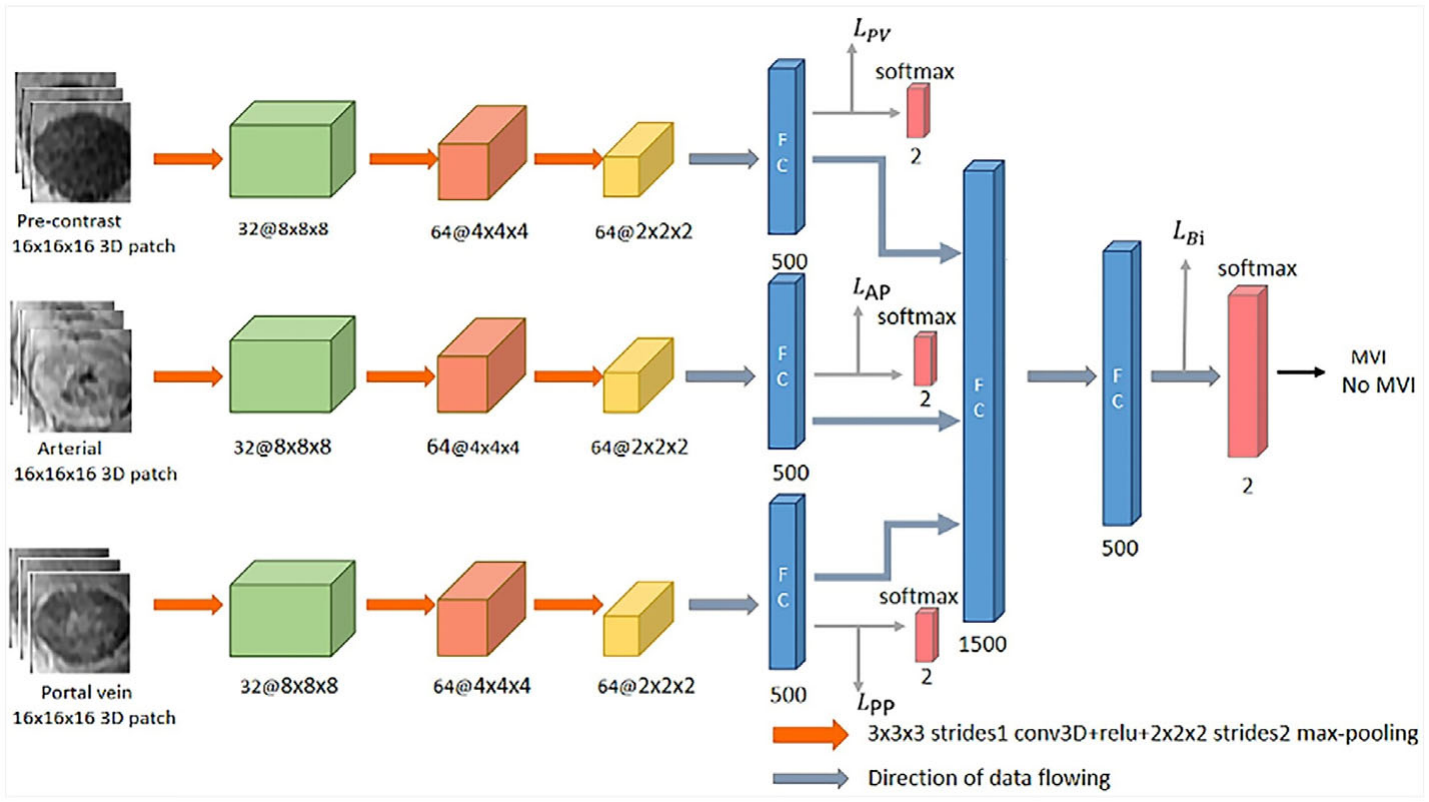
The flowchart of the proposed deep learning framework.

**Figure 2** shows the designed network structure, which uses 3D CNN to extract deep features related to MVI characterization from contrast-enhanced MR, and the loss function of deep supervision is designed to better predict MVI. First, many 3D block samples (16×16×16) were extracted in the tumor area *via* image resampling method in the three phases of contrast-enhanced MR. Then, the spatially corresponding 3D deep features are extracted from those 3D block samples for MVI prediction based on the 3D CNN. Subsequently, the 3D deep features from the three phases are concatenated, and connected with two fully connected layers and softmax layer for the final classification. Finally, the loss function for deep supervision is designed to combine the three loss functions corresponding to the pre-contrast, arterial and portal vein phases and the loss function related to the concatenated 3D deep features. The following sections will introduce the details of the designed network structure.

#### 3D CNN

In this work, the 3D CNN architecture which is a straightforward extension of typical 2D CNN architecture (28) is used to extract the 3D deep features of each phase in the contrast-enhanced MR images for MVI prediction of HCC. In detail, there are typically several convolutional layers, pooling layers, fully connected layers and a softmax layer in the 3D CNN architecture. For the convolutional layer, a 3D convolution operation (3×3×3) is performed on the extracted 3D block samples (16×16×16) to obtain the convolution feature map. The pooling layer is performed by image downsampling to reduce the size of 3D block samples in order to obtain different scales of deep features. In the fully connected layer, the neuron is connected to all the activations in its previous layer, which is mainly used to reduce the dimensionality of the acquired deep features. After the output of the last fully connected layer is connected to the “softmax” layer, the classification result will be output.

#### Loss Function

The deep supervision network (DSN) was originally proposed to directly supervise the features of the hidden layer and improve the effect of the hidden layer on the final performance during the CNN learning process (29). In this work, we expect to directly supervise the 3D depth features from the three phases of contrast-enhanced MR, thereby constraining the feature learning process of each phase to improve classification performance. The cross-entropy is adopted as the loss function for the concatenated features and the deep features corresponding to the three phases. The loss function for the concatenated features is defined as follows:

1 $$L_{CON}=-\sum_{k}y_{k}^{’}\log\;(y_{k})$$

where yʹ is the ground-truth label of MVI information, and y is the output probability of MVI prediction by the CNN. Therefore, the deeply supervised loss function is the summation of the cross-entropy loss function of the concatenated feature and the cross-entropy loss functions of all deep features corresponding to each phases of contrast-enhanced MR, which can be defined as follows:

2 $$\iota =L_{CON}+L_{PP}+L_{AP}+L_{PV}$$

where L_PP_, L_AP_, and L_PV_ are the supervised loss functions of 3D CNN corresponding to the three phases of contrast-enhanced MR, respectively.

#### The Implementation

The proposed network structure was implemented by “Tensorflow” (https://tensorflow.google.cn/install), and training and testing were performed under the configuration of GeForce GTX1080 8G. The optimization process of the total loss function of the deep network used the Adam algorithm (30). In order to improve the generalization performance of the network and reduce the risk of overfitting, we also adopted the “dropout” (31) mechanism and the parameter was set to 0.5. In addition, the neuron activation function “ReLU” (32) was used to accelerate the convergence of the network. The parameters of the network layer were set as follows: the size of the 3D convolution filter was 3×3×3, the stride was 1, the maximum buffered kernel size was 2×2×2, the stride was 2, and the learning rate was initialized to 1e−4, the attenuation of the learning rate was 0.98.

### Statistical Analysis

For numerical variables, independent student’s t test or Mann-Whitney U test was used, and for categorical variables, chi-square test, or Fisher’s exact test was used to evaluate the statistical difference of age, gender, and HCC tumor diameter between MVI and no MVI. In order to evaluate the stability of the learning network and reduce measurement errors, the training and testing process were repeated five times. Accuracy, sensitivity, and specificity were expressed as the mean ± standard deviation of five repeated measurements in the test set. Note that MVI positive corresponds to the positive class, so sensitivity measured the ability of the proposed model to detect MVI positive of HCCs, while specificity measured the ability to differentiate MVI negative of HCCs. The output probability value of the deep learning model in the test set in differentiating the MVI present and MVI absent was evaluated by independent student’s t-test. Receiver operating characteristic curve (ROC) analysis was adopted to evaluate the performance in predicting the MVI. P<0.05 was considered statistically significant. Computer software packages (R software, version 3.6.1) were used for the statistical analyses.

## Results

### Training and Validation Dataset

Among the 117 lesions, 70 were pathologically determined as the absence of MVI, while 47 were pathologically determined as the presence of MVI. In order to verify the performance of the deep learning model for MVI prediction, the dataset was divided into two parts, including 77 HCCs for the training dataset and the remaining 40 HCCs for the independent validation dataset. **Table 2** summarized the clinical characteristics of patients in training and validation cohorts, in which 30 MVI positive lesions and 47 MVI-negative lesions were chosen as the training set.

### Performance of Clinical Information

As shown in **Table 2**, it can be found that tumor size, α-fetoprotein level, and presence of hemorrhage have statistical significance to differentiate the MVI present and absent in both training and validation cohorts. The performance of the three clinical variables in the test cohort with cutoff values determined in the training cohort was shown in **Table 3**. The AUC values of the three clinical variables were 0.715, 0.705, and 0.664, respectively. The AUC value of three-variables-model refers to nodules long diameter + presence of hemorrhage + α-fetoprotein level was 0.798.

**Table 3:** Performance of three clinical variables in the test cohort whereas cutoff is determined in the training cohort using Youden Index.

Characteristics	Accuracy	Sensitivity	Specificity	AUC	Cutoff
Nodules long diameter	70%	70.59%	69.57%	0.715 (95% CI: 0.549–0.881)	43mm
α-Fetoprotein level	60%	82.35%	43.48%	0.705 (95% CI: 0.527–0.882)	14ng/ml
Presence of hemorrhage	67.50%	58.82%	73.91%	0.664 (95% CI: 0.512–0.815)	0.5*
Combination	72.50%	64.71%	78.26%	0.798 (95% CI: 0.649–0.947)	0.458*

*The cutoffs refer to prediction probability determined by logistic regression model.

### Performance of Deep Learning Model

**Figure 3:**
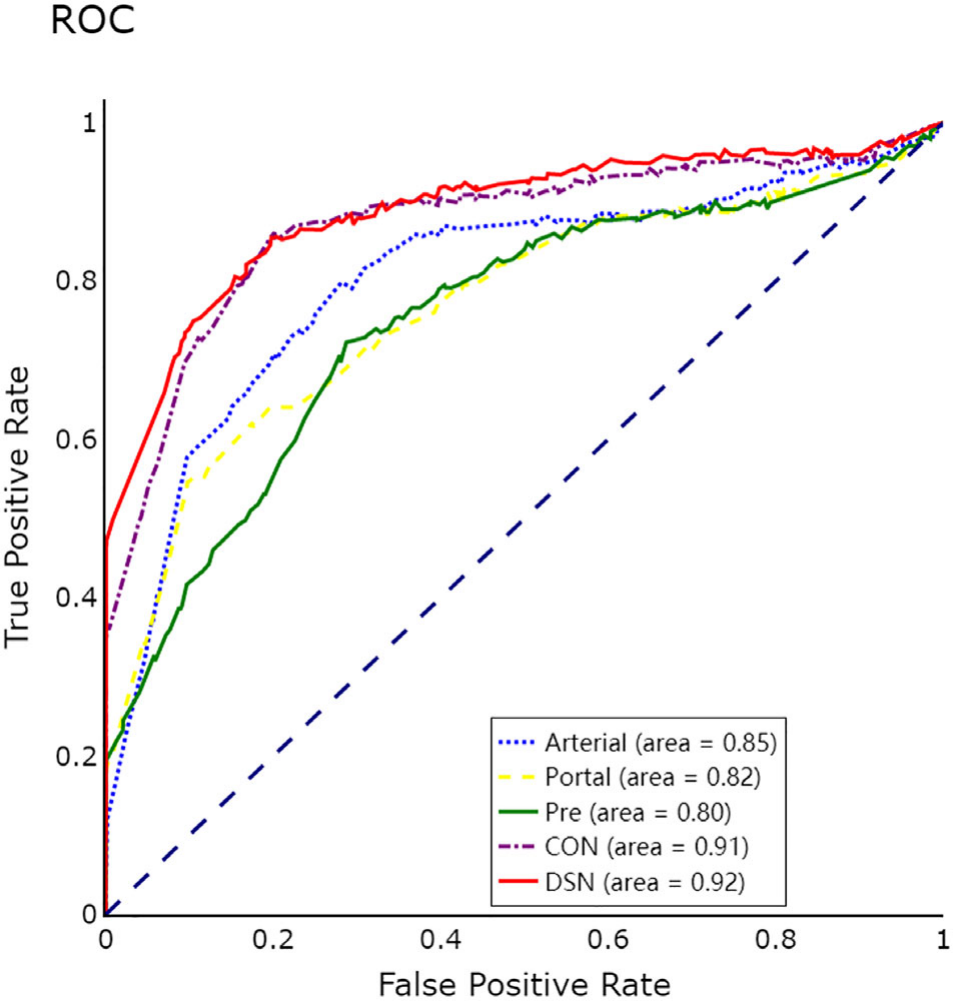
ROC curves of 3D convolutional neural networks (CNN) for microvascular invasion (MVI) prediction in single phases and multiple phases.

With respect to each phase of contrast-enhanced MR, we separately assess the performance of 3D CNN for MVI prediction in different phases. As tabulated in **Table 4**, it could be found that the 3D deep features of the arterial phase had the best performance in predicting MVI, which was better than the portal phase and the pre-contrast phase. In addition, compared to the single phase used for MVI prediction, the 3D deep feature concatenation (CON) method (33) from pre-contrast, arterial, and portal vein phases further improved performance. Clearly, the proposed method of 3D deep feature fusion with deep supervision net (DSN) yielded best performance. Finally, **Figure 3** also plotted the ROC curves based on 3D CNN for single-phase and multi-phase combination for MVI prediction.

**Table 4:** Performance of microvascular invasion (MVI) prediction using 3D convolutional neural networks (CNN) in single phases and the combination of multiple phases (%).

Framework	Accuracy	Sensitivity	Specificity	AUC	p-value
Pre-contrast	72.00 ± 1.00	66.25 ± 5.00	75.83 ± 4.86	79.33 ± 1.67	0.001
Arterial	80.00 ± 1.58	80.00 ± 7.29	80.00 ± 3.12	85.47 ± 1.81	0.000
Portal vein	74.00 ± 1.22	71.25 ± 5.00	75.83 ± 1.67	81.72 ± 2.53	0.000
Concatenation	85.00 ± 1.58	86.25 ± 6.12	84.17 ± 3.12	90.57 ± 2.66	0.000
Proposed DSN	87.50 ± 1.58	86.25 ± 4.68	88.33 ± 3.12	92.55 ± 1.71	0.000

### Detailed Study of the Deep Learning Model

**Figure 4:**
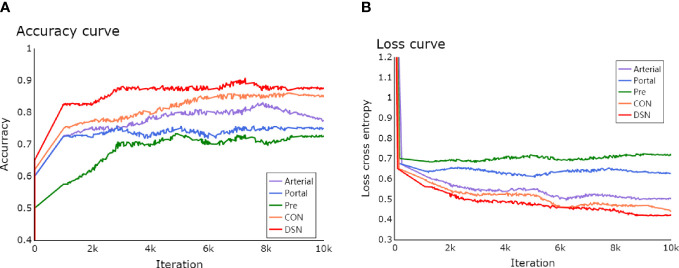
Test loss and accuracy **(A)** curves for different iterations **(B)**.

**Figure 5:**
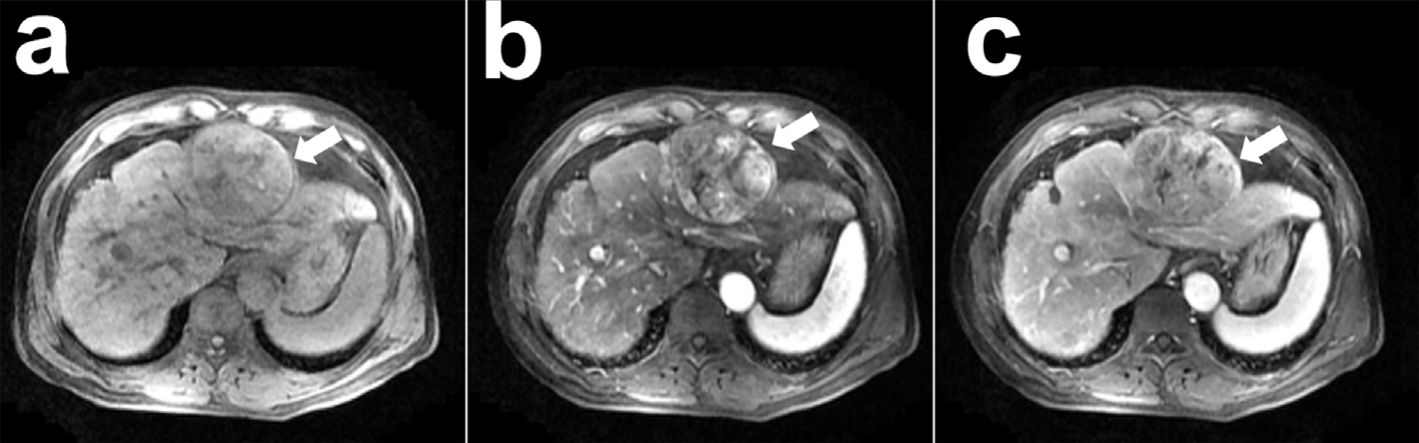
A case of hepatocellular carcinoma (HCC) with contrast-enhanced MR: a 51-year-old man with pathological confirmed HCC (white arrow) and microvascular invasion (MVI) present. This neoplasm was misdiagnosed as the absence of MVI by the 3D CNN model with pre-contrast phase **(A)**, arterial phase **(B)**, portal vein phase **(C)** images and concatenation (CON), while the proposed 3D convolutional neural networks (CNN) with deep supervision net (DSN) model made correct diagnose as the present of MVI.

The learning process of the framework with limited number of clinical HCCs for MVI prediction was shown in detail. In **Figure 4**, the total loss function curve and its corresponding accuracy curve were shown for the test data. We could find that the test loss was significantly reduced after iterations, which indicated that the proposed deep learning framework had been successfully optimized. In particular, the values of test loss were gradually reduced, and the values of test accuracy were continuously improved. Even if the amount of training tumor sample data was small, the problem of network overfitting was not observed. **Figure 5** showed a case of HCCs in which the single models were misdiagnosed, and the proposed fusion model made correct decisions.

## Discussion

In the present research, we propose MVI prediction based on 3D CNN and contrast-enhanced MR images. This deep learning framework may have the potential in use for the precise medicine of neoplasm, especially in the application of small data for lesion characterization. To evaluate the performance of each phase, our experimental results show that the MVI produced in the arterial phase has the best predictive performance, which is better than the portal vein phase and the pre-contrast phase. The possible explanation might be that the arterial phase of contrast-enhanced MR images embedded MVI information of vascularity and cellularity of HCC, which can be well characterized by the deep learning framework with 3D CNN.

In this study, we have applied the 3D CNN for MVI prediction rather than the conventional 2D CNN. In fact, conventional 2D CNN has recently been used for liver fibrosis staging (23) and liver mass diagnosis (24). Since 2D CNN is based on a single slice and ignores the spatial information of the third dimension, 3D CNN is a better solution to characterize tumors, which can take advantage of the three-dimensional spatial information in volumetric data to more accurately characterize the lesion (34, 35). Recently, Hamm CA et al. (36) used 3D CNN to classify 494 lesions from 6 types of liver tumor entities on multiphase MRI, and demonstrated the high performance of 3D CNN for lesion characterization.

Furthermore, our study also investigates the performance of MVI prediction using 3D CNN with multiple phases of contrast-enhanced MR. This study shows that deep feature fusion from multiple phases of contrast-enhanced MR can significantly improve the prediction performance of MVI. Conventional multimodal fusion method with simple concatenation of 3D deep features (33) outperforms the performance of MVI prediction using 3D CNN in single phases, while our proposed method of 3D deep feature fusion with DSN exhibits even better performance than that of the simple concatenation. Deep supervision loss function that integrates loss functions of multiple phases and combines the three-loss functions corresponding to multiple phases can yield the best performance of MVI prediction. To our knowledge, very few studies had considered multi-modality medical information for better MVI prediction, while the proposed framework can simultaneously make use of multiple phases of contrast-enhanced images for better MVI prediction.

Radiomics features have been widely used as the non-invasive prognosis or predictive biomarker for MVI prediction. Peng et al. (11) proposed an imaging radiomics model to predict the risk of MVI by analyzing preoperative contrast-enhanced CT images and obtained the performance with the AUC value of 0.844 in the validation cohort. Feng et al. (13) proposed a radiomics model to analyze the hepatobiliary phase in Gd-EOB-DTPA-enhanced MRI for preoperative MVI prediction with the AUC value of 0.85. Xu et al. (15) recently assessed the radiomics characteristics for MVI prediction, and demonstrated that the radiomics signatures of contrast-enhanced CT were less important than the radiological features, with the AUC value from 0.787 to 0.841. In addition, the radiomics nomogram has also been used in the ultrasound images for MVI prediction with the reported AUC value of 0.731 (16) and 0.806 (17), respectively. Comparatively, our proposed deep learning framework with 3D CNN yielded the performance with the AUC value of 0.926, which is better than the reported radiomics approaches. It should be noted the present study is totally different from the radiomics approach. First, different medical images were used for the assessment of MVI prediction. Second, our proposed deep learning method with 3D CNN is based on deep feature extracted from 3D patches from lesions, while the radiomics approach is often based on radiomics features extracted from 2D region of interest in tumors. Third, our proposed deep learning method with 3D CNN assessed the performance of MVI prediction in single phases as well as the combination of multiple phases, while the radiomics approach is generally conducted in single phases.

It has been reported that tumor size has a certain effect on predicting MVI (37). In the present study, we find that there is statistical significance of tumor size for MVI prediction. We also used ROC analysis to calculate the predictive performance of tumor size for MVI. However, its performance for MVI prediction is not high enough (AUC=0.715, 95% CI: 0.549–0.881), which is much lower than our proposed deep learning framework. In addition, performance of clinical information has been comparatively assessed for MVI prediction with the AUC value of 0.798 (**Table 3**) in this study, which exhibits relatively lower performance than the deep learning method. The combination of clinical characterization and radiomics feature was shown to further improve the prediction performance (14, 15). Therefore, the combination of 3D deep features and clinical features may be expected to achieve better MVI prediction.

This present study does not assess the performance of 3D CNN in the delayed phase as 25 of the collection of clinical data fluctuate in the delay period (1–3 min) and different positions (coronal). In addition, we do not suggest that the delayed phase yields promising results for MVI prediction. As the contrast agent has overflowed from the tumor region in the delayed phase, the tumor region becomes dark and the tissue cellularity and vascularity within the tumor become to be unclear. Therefore, the heterogeneous of intensity distribution within the tumor may not be precisely represented by the deep feature.

There are several limitations to this retrospectively study. First, the data set was collected in a single institution, and the number of HCCs used in this study is limited. Although 1,114 HCCs were retrieved from the database, only 117 met the inclusion criteria of this study. Large multicenter studies and more samples are required to assess the predictive performance of the deep learning framework precisely. Secondly, we did not consider the influence of MR data slice thickness on prediction performance. Since a larger slice thickness will affect the image quality of the 3D block, future work will consider the influence of slice thickness on the prediction performance of 3D CNN. Third, simple image resampling is used for data augmentation to increase the number of training sets. This is the most common way of deep learning for small clinical samples. However, image patches may contain large overlapping areas with homogeneous features, which may result in a high risk of over-fitting for the deep learning framework. More advanced data augmentation methods, such as generative adversarial network (38), are expected to increase the discrepancy of augmented samples. Furthermore, other contrast agents or hepatobiliary phase with Gd-EOB-DPTA enhanced MR imaging, have not been comparatively evaluated in the present study. Finally, we only derive 3D deep feature from contrast-enhanced MR images for MVI prediction in this study. Embedding clinical information and radiological features into the current deep learning framework for better MVI prediction will be an important work in the future.

In conclusion, we propose a learning network based on 3D CNN and contrast-enhanced MR for MVI prediction, which extracts discriminative features from each phase of contrast-enhanced MR and combines them to obtain better prediction results. Although the current purpose of this task is to predict MVI, we believe that the proposed framework can be widely used in the description of many lesions in clinical practice.

## Data Availability Statement

All datasets presented in this study are included in the article/supplementary material.

## Ethics Statement

The studies involving human participants were reviewed and approved by Guangdong General Hospital, Guangdong Academy of Medical Sciences. The patients/participants provided their written informed consent to participate in this study.

## Author Contributions

WZ: study concept, image preprocessing, experimental design, writing of manuscript, and funding. WJ and HG: experimental design and test. XC: data analysis and statistical analysis. LZ, ZL, and CL: study concept and data analysis. GW: study concept, data collection, experimental design, and editing the manuscript, funding. All authors contributed to the article and approved the submitted version.

## Funding

This research is sponsored by the grants from National Natural Science Foundation of China (81771920) and the Key R&D Program of Guangdong Province of China (2018B030339001).

## Conflict of Interest

The authors declare that the research was conducted in the absence of any commercial or financial relationships that could be construed as a potential conflict of interest.
